# A Study on the Influence of Speed on Road Roughness Sensing: The SmartRoadSense Case †

**DOI:** 10.3390/s17020305

**Published:** 2017-02-07

**Authors:** Giacomo Alessandroni, Alberto Carini, Emanuele Lattanzi, Valerio Freschi, Alessandro Bogliolo

**Affiliations:** DiSPeA—University of Urbino, 61029 Urbino, Italy; g.alessandroni2@campus.uniurb.it (G.A.); emanuele.lattanzi@uniurb.it (E.L.); valerio.freschi@uniurb.it (V.F.); alessandro.bogliolo@uniurb.it (A.B.)

**Keywords:** SmartRoadSense, collaborative monitoring, road roughness index

## Abstract

SmartRoadSense is a crowdsensing project aimed at monitoring the conditions of the road surface. Using the sensors of a smartphone, SmartRoadSense monitors the vertical accelerations inside a vehicle traveling the road and extracts a roughness index conveying information about the road conditions. The roughness index and the smartphone GPS data are periodically sent to a central server where they are processed, associated with the specific road, and aggregated with data measured by other smartphones. This paper studies how the smartphone vertical accelerations and the roughness index are related to the vehicle speed. It is shown that the dependence can be locally approximated with a gamma (power) law. Extensive experimental results using data extracted from SmartRoadSense database confirm the gamma law relationship between the roughness index and the vehicle speed. The gamma law is then used for improving the SmartRoadSense data aggregation accounting for the effect of vehicle speed.

## 1. Introduction

Several studies—starting in the late 1950s—have shown that road surface quality is the most important criteria for the evaluation of a road path and its drive comfort. The deterioration of roads leads to more expensive maintenance, not only for the road itself but also for vehicles. Every vehicle increases its operating costs: first, with increased fuel consumption and, most importantly nowadays, with more emissions to the environment; second, with mechanical failures at suspensions and other car components. At the same time, the additional dynamic load of vehicles causes more pavement failures [1,2,3].

Consolidated approaches for monitoring road surface conditions involve the adoption of costly and sophisticated hardware equipments such as laser profilers [4] or specific accelerometers with data acquisition systems [5], whose cost, for each car (also taking into account calibration and installation), can be significant.

In the attempt to reduce the road monitoring cost, in the literature, many studies have explored the use of standalone accelerometers or accelerometers embedded in mobile devices to detect bumps, potholes, bumpy roads. In [6], a standalone accelerometer mounted in a car is used to assess the road conditions. It is shown that the road roughness can be estimated from the acceleration data obtained from the sensor. In [7], a standalone accelerometer with high sample frequency (380 Hz) is used to detect the road anomalies, using a cascade of filters for separating different classes of events. In [8], several sensing components of the mobile device, e.g., accelerometer, microphone, GSM radio, and GPS, are used to monitor road and traffic conditions. By analyzing the sensors data, potholes, bumps, braking and honking are detected. Similarly to [7], an accelerometer with high sampling frequency (of 310 Hz, a frequency higher than that of most accelerometers embedded in current mobile devices) is used in [8] for detecting potholes or bumps. Smartphones equipped with accelerometers are used in [9,10] for detecting the location of potholes, monitoring events in the acceleration data. In [11], the smartphone accelerometer data is used to detect braking events and bumps on roads using machine learning techniques, i.e., K-means clustering and support vector machines. Smartphone accelerometers and gyroscopes are used in [12,13] to detect road surface anomalies, using the stationary wavelet transformation decomposition for signal analysis. Mobile devices equipped with accelerometers are also used in [14,15]. The accelerometer data are analyzed in the frequency domain to extract features corresponding to road bumps. Smartphone accelerometers are used to detect bumps, potholes, manhole covers, and other anomalies in [16,17]. Mohamed et al. investigate the use of the gyroscope data around gravity rotation to detect speed bumps [18]. Mukherjee et al. describe a method of detection of road bumps using the accelerations recorded by smartphones. The quarter car and half car models are used to develop signatures of both single and multiple bumps. The records of smartphones are filtered and compressed to highlight the effect of bumps and it is shown that signature of bumps can be clearly seen in the recordings [19]. In [20], the vertical component of the smartphone accelerometer readings is extracted with a signal processing heuristic. Then, road anomalies such as potholes and dumps are detected with an heuristic based on the underdamped oscillation model. To account for different vehicle velocities, the detection parameters are changed according to the vehicle speed. A road defects detection system exploiting a mobile data collection kit mounted on vehicles is introduced in [21]: accelerometer data are recorded only when some root-mean-square thresholds are passed and data are sent to a back-end server, where a supervised machine learning technique is applied to classify the data fragments in different categories.

The majority of these studies focus mainly on identifying and locating singles anomalies (or classes of anomalies). On the contrary, more recent research projects [22,23,24,25,26,27,28,29,30,31] have focused on using the accelerometer data measured by smartphones or other mobile devices for estimating a roughness index, characterizing the road surface status. In [22], a low cost hardware module embedding low-end accelerometers and GPS devices is used detect road potholes and evaluate road roughness levels. The potholes detection is performed exploiting Gaussian mixture models and the international roughness index (IRI) is estimated using the empirical formula of [32].

Machine learning techniques are used also in [30], where the authors use smartphones sensor data to estimate the international roughness index of the travelled roads by training a feature-based prediction model and exploiting ground truth information of the road condition from official measurements. In [31], the mean-absolute-value of the vertical acceleration sensed by a smartphone travelling on a motorbike is computed every 100 meters. The data are sent to a server where a fuzzy classifier deduces the road roughness. In [25] the data of accelerometer is compared with the IRI previously measured on the same road under assessment. The accelerometer samples are first highpass filtered in order to eliminate or reduce the effect of gravity acceleration, vehicle accelerations, and centrifugal accelerations at curves. Then the filtered samples are analyzed in the frequency domain in different bands. It is shown that the magnitude of the spectrum is approximately proportional to the road IRI. A similar but different approach is used in SmartRoadSense [27]. In [27] the smartphone accelerometer samples are segmented on 1s-length frames. Linear predictive coding (LPC) analysis is performed on the accelerometer signals on a frame by frame basis and the average power of residual error is used to estimate a roughness index. The LPC analysis removes the influence of gravity acceleration, vehicle accelerations, and centrifugal accelerations at curves, roll, pitch, and yaw accelerations due to road trend, and vibrations due to the engine. Indeed, the LPC analysis is able to remove all predictable slowly varying or periodic components of the signal. The LPC residual preserves the unpredictable part of the accelerations, which can be mostly related to the effect of the road surface on the car through tires and suspensions. The GPS coordinates, the vehicle speed and the roughness index are periodically transmitted to a data center, which aggregates the information coming from different mobile devices compensating for their different sensitivities [33]. Eventually, the resulting data are stored in a Geographical Information System (GIS) where each point is computed as a weighted average, over time and space, of the data points mapped to the same road. After an initial study and testing period, since 21 February 2015, the SmartRoadSense application has been officially released and it has become fully operative. In nine months, SmartRoadSense has collected more than 24,000 km, which correspond to about 5% of the entire Italian road network [26,34]. The SmartRoadSense database contains the roughness indexes of these roads, 5,800,000 samples of roughness indexes extracted by 1,750,000,000 values of accelerations, obtained from 432 different vehicles using 147 different models of mobile devices. Some Italian municipalities are currently adopting the SmartRoadSense system. It is expected that large scale adoption will provide useful information on the capability of the platform to evaluate the quality of monitored roads surface.

This paper studies the influence of the vehicle speed on the vertical acceleration sensed by the mobile device in the car cabin and consequently the influence on the roughness index of SmartRoadSense. An ideal point closely following the road profile with constant horizontal speed senses a power of vertical acceleration that is proportional to the fourth power of its speed [35]. Thus, the vehicle speed is an important parameter whose effect should also be accounted for when processing the accelerometer data or when aggregating data coming from different mobile devices. The influence of speed on the accelerometer data is however much different from that observed considering an ideal point. Indeed, the accelerometer embedded into a mobile device senses the road elevation profile through the tires, the suspension system, and the mechanical coupling with the car cabin. The response of these mechanical parts influences the dependence on speed. In this paper, using a theoretical model of the road profile and of the car tires and suspensions, it is shown that a mobile device anchored to the car cabin senses a power of vertical acceleration that can be locally related to the vehicle speed according to a gamma law. It is also shown that the same gamma law relates the roughness index of SmartRoadSense to the vehicle speed. The theoretical results are confirmed by experiments performed with different mobile devices using the data collected within SmartRoadSense project. The experimental results also highlight that the gamma law depends on the type of road, i.e., asphalt. Different gamma laws are obtained for motorway, trunk, primary, and secondary roads. The gamma laws estimated from the database for each kind of road can then be used for improving the SmartRoadSense data aggregation, accounting for the effect of vehicle speed.

The rest of the paper is organized as follows. In Section 2, using a theoretical model of the road surface of the car suspensions, it is shown that vertical acceleration sensed by the smartphone depends on the vehicle speed according to a gamma law. The SmartRoadSense architecture and analysis algorithm are summarized in Section 3 where it is shown that the roughness index depends on the vehicle speed according to same gamma law. Section 4 provides experimental results using the data collected within SmartRoadSense project. Section 5 explains how the measured gamma laws can be used for improving data aggregation and provides experimental results illustrating the effect of the correction. Eventually, conclusions follow in Section 6.

## 2. A Study on the Vertical Acceleration Sensed in the Car Cabin

In this section a theoretical study of the vertical acceleration sensed by the smartphone and due to the road roughness in presented. Our purpose is to find the relationship between the vertical acceleration sensed in the car cabin and the vehicle speed. In what follows we first review the models of the road surface and of the car suspensions adopted in the study. Then, with these models we study the relationship between the power of vertical acceleration in the car cabin and the vehicle speed.

### 2.1. Road Surface Model

Different models of road elevation profile have been proposed in the literature. The road elevation profile has been first modeled using step functions, triangular waves, or sine waves [36] and, in more accurate studies [35], as the sum of sine waves with randomly generated sinusoidal functions with different amplitudes and phases. In [35], it was also shown that the spatial power spectral density (PSD) of a typical road surface has a low–pass characteristic, which decreases at the increase of the spatial frequency. This observation has led to a well accepted model of the road elevation profile, as a white Gaussian noise filtered by a first order low–pass filter [35,37]. Other stochastic models with low-pass characteristics have been recently proposed to describe the randomness of measured road profiles, such as non-homogeneous Gaussian processes [38], homogeneous Laplace moving average (LMA) processes and hybrid combinations [39]. However, the Gaussian model of [35] remains one of the most popular road models for its ability to represent “undamaged” road surfaces [39]. Thus, in our study, we assume the road elevation profile w(x) can be modeled as white Gaussian noise filtered by a first order low–pass filter. The white Gaussian noise has autocorrelation function $\rho_{ww}\left(x\right)=q\delta \left(x\right)$, and PSD $S_{ww}\left(\Lambda \right)=q$, where *q* is the PSD magnitude, δ(x) is the Dirac delta function, and Λ is the angular wavenumber measured in radiants per meter. The first order low–pass filter has angular wavenumber response

(1) $$H\left(j\Lambda \right)=\frac{1}{p+j\Lambda },$$

with *p* the low–pass filter pole.

The spatial PSD of the road elevation profile $S_{rr}\left(j\Lambda \right)$ is given by

(2) $$S_{rr}\left(j\Lambda \right)=S_{ww}\left(j\Lambda \right){\left|H(j\Lambda )\right|}^{2}=q{\left|\frac{1}{p+j\Lambda }\right|}^{2}.$$

In this model, the statistical properties of the road profile are completely characterized by parameters *q* and *p*.

Let us consider an ideal point closely following the road profile and moving with constant horizontal speed *v*. Ndoye et al. proved that, from (2), the PSD of the vertical acceleration $A_{y}$ of this ideal point has continuous time Fourier transform given by

(3) $$S_{A_{y}A_{y}}\left(j\Omega \right)=q{\left|\frac{{\left(j\Omega \right)}^{2}}{p+j\frac{\Omega }{v}}\right|}^{2},$$

where Ω is the angular frequency in radiants per second [37] .

The road parameters *q* and *p* of (2) can also be obtained by analyzing the PSD of vertical acceleration [37].

In the SmartRoadSense application, the vertical acceleration is sensed with an accelerometer embedded in a mobile device anchored inside the car cabin. The accelerometer senses the road elevation profile through tires, suspensions, and the mechanical coupling with the car cabin. Thus, the PSD in (3) is sensed only after passing through all mechanical parts.

### 2.2. Quarter-Car Model of Suspensions

**Figure 1:**
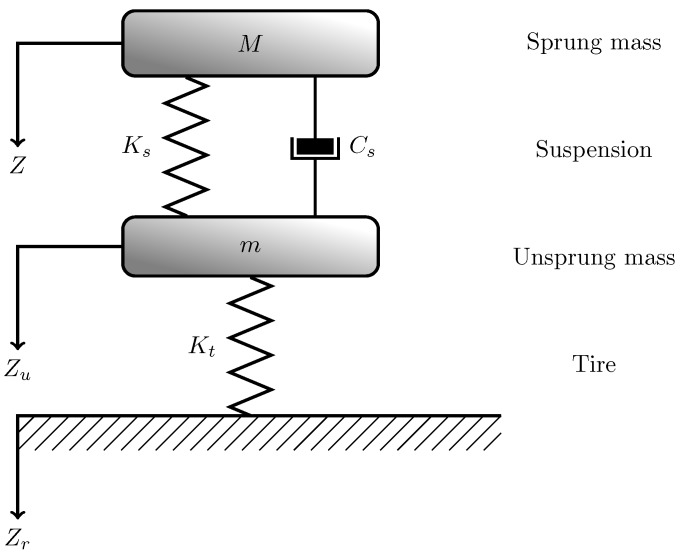
Mathematical model of quarter-car.

At the most basic level, each vehicle has an insulation from the ground implemented by the suspension system connected to each tire. The suspensions are the system of springs and shock absorbers connected to the tires that allow the car to move smoothly with reduced shocks to its occupants. These suspensions aim to reduce the vertical acceleration of the sprung mass when the vehicle travels over a road with roughness. The essential dynamic of the suspension model is shown in Figure 1. The tire can be represented by a single spring, although in some cases a damper is included to represent the damping contribution due to its viscoelastic nature [1,2]. The suspensions are represented with a spring equipped with stiffness and damping.

In Figure 1, *M* is the sprung mass (above the suspension system) and *Z* is its level. $K_{s}$ is the stiffness and $C_{s}$ is the damping constant of the suspension that connects *M* to *m*, where *m* is the unsprung mass (above the tire and below the suspension) and $Z_{u}$ is its level. $K_{t}$ is the stiffness of the tire. Eventually, $Z_{r}$ is the ground level. The model, which is related to a single suspension, is known as “Quarter-car model” [40]. The suspension system has a mathematical model which can be solved using the Newton’s second law.

**Figure 2:**
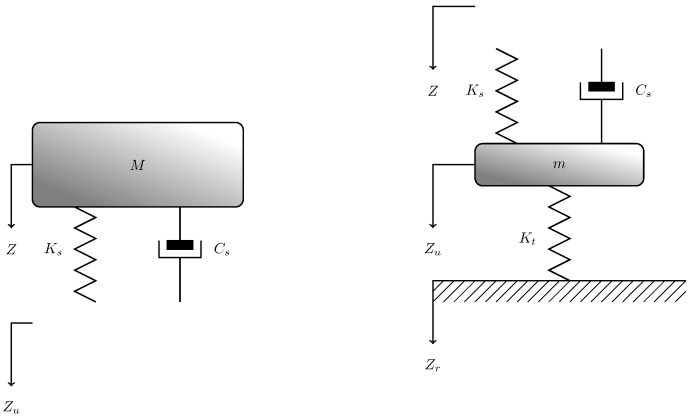
Mathematical model of quarter-car broken down in two parts.

The force applied to each mass *M* and *m* can be derived by decomposing the quarter-car model in two parts as in Figure 2.

By the analysis of the two parts of the model, the following 2-nd order differential equations are derived,

(4) $$\left[\begin{array}{c} M\\ m \end{array}\right]\left[\begin{array}{c} \overset{..}{Z}\\ \overset{..}{Z_{u}} \end{array}\right]+\left[\begin{array}{cc} C_{s} & -C_{s}\\ -C_{s} & C_{s} \end{array}\right]\left[\begin{array}{c} \dot{Z}\\ \dot{Z_{u}} \end{array}\right]+\left[\begin{array}{cc} K_{s} & -K_{s}\\ -K_{s} & K_{s}+K_{t} \end{array}\right]\left[\begin{array}{c} Z\\ Z_{u} \end{array}\right]=\left[\begin{array}{c} 0\\ K_{t}Z_{r} \end{array}\right],$$

where $\dot{Z}$ and $\overset{..}{Z}$ are respectively the first and second derivatives of *Z* with respect to the time *t* in the Newton’s notation.

Using (4), the frequency response of the system that relates the vertical acceleration in the car cabin to the vertical acceleration at ground level caused by the road profile can be computed,

(5) $$S\left(j\Omega \right)=\frac{Dj\Omega +1}{A{\left(j\Omega \right)}^{4}+B{\left(j\Omega \right)}^{3}+C{\left(j\Omega \right)}^{2}+Dj\Omega +1},$$

where

$$A=\frac{\chi }{K_{1}K_{2}},$$

$$B=c\frac{1+\chi }{K_{1}K_{2}},$$

$$C=\frac{K_{1}+K_{2}\chi +K_{2}}{K_{1}K_{2}},$$

$$D=\frac{c}{K_{2}},$$

and $\chi =\frac{m}{M}$, $c=\frac{C_{s}}{M}$, $K_{1}=\frac{K_{t}}{M}$, and $K_{2}=\frac{K_{s}}{M}$. Different typical values of $K_{s}$, $K_{t}$, $C_{s}$, *M*, and *m* have been suggested in the literature [35,41,42,43,44,45,46]. Exploiting these parameters, the coefficients of the quarter-car transfer functions *A*, *B*, *C*, and *D* reported in Table 1 have been computed.

**Figure 3:**
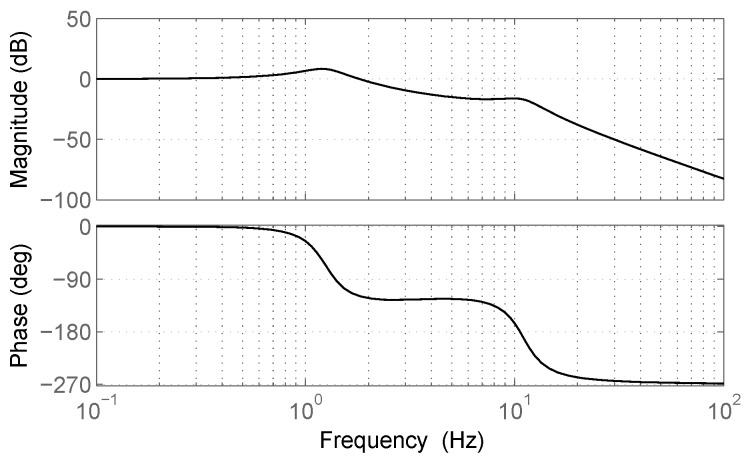
Frequency response of the system that relates the vertical acceleration in the car cabin to the vertical acceleration at ground level according to the quarter-car model.

Table 1 shows that all parameters (with the exceptions of Allison [45]) have the same order of magnitude. Thus, the parameters give very similar frequency responses. In the following, for simplicity sake we will refer to the coefficients *A*, *B*, *C*, and *D* computed with the parameters provided by Gillespie ([35] Figure 5.25, p. 163). The resulting frequency response is reported in Figure 3. The system has the behavior of a low-pass filter with a cut-off frequency around 2 Hz.

### 2.3. The Power of Vertical Acceleration

Using the Gaussian model of the road surface and the quarter-car model of suspension, we can now estimate the power of vertical acceleration P(v) sensed in a vehicle moving with constant horizontal speed *v*. Applying the Parseval theorem, we obtain

(6) $$P\left(v\right)=\frac{1}{2\pi }\int_{-\infty }^{+\infty }S_{A_{y}A_{y}}\left(j\Omega \right){\left|S\left(j\Omega \right)\right|}^{2}d\Omega ,$$

where $S_{A_{y}A_{y}}\left(j\Omega \right)$ is the PSD in (3) which depends on the speed *v*, and S(jΩ) is given by (5). The integral can be computed with the method of adaptive quadrature [47].

**Figure 4:**
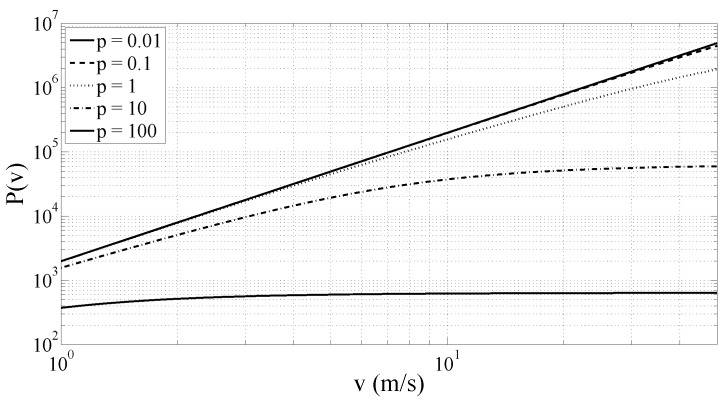
Power of vertical acceleration for different values of the pole *p*.

Figure 4 shows the behavior of P(v) for five different orders of magnitude of pole *p*. For low values of *p* or for low speed values, P(v) increases by 20 dB/decade. On the contrary, when *p* or *v* become very large, P(v) tends to a constant. This behavior can also be explained from (3). Let us consider $\hat{p}=pv$, such that

(7) $$S_{A_{y}A_{y}}\left(j\Omega \right)=q{\left|v\frac{{\left(j\Omega \right)}^{2}}{\hat{p}+j\Omega }\right|}^{2}.$$

When $\hat{p}\ll j\Omega$, in (3) we have

(8) $$S_{A_{y}A_{y}}\left(j\Omega \right)=q{\left|v\frac{{\left(j\Omega \right)}^{2}}{\hat{p}+j\Omega }\right|}^{2}=v^{2}q\Omega^{2}.$$

On the contrary, when $\hat{p}\gg j\Omega$,

(9) $$S_{A_{y}A_{y}}\left(j\Omega \right)=q{\left|v\frac{{\left(j\Omega \right)}^{2}}{\hat{p}+j\Omega }\right|}^{2}=\frac{q}{p^{2}}\Omega^{4}.$$

Introducing these results in (6), and since *q* and S(jΩ) are independent of *p* and *v*, it follows that when $\hat{p}$ tends to 0, P(v) is proportional to $v^{2}$, while when $\hat{p}$ tends to infinity, P(v) is proportional $v^{0}$, i.e., is independent of *v*.

**Figure 5:**
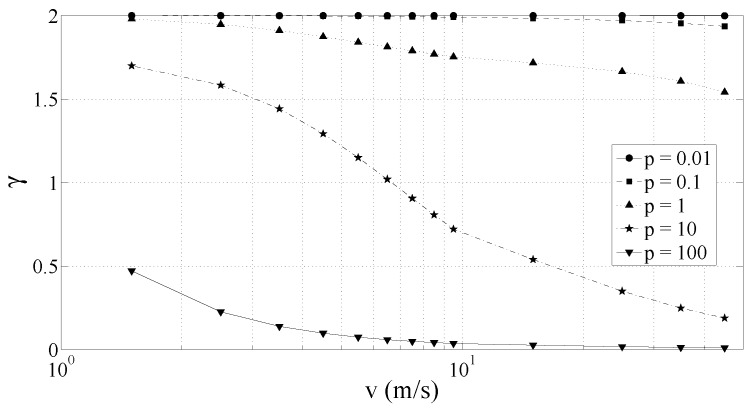
*γ* coefficient versus speed for different values of the pole *p*.

Locally, P(v) can be approximated with a gamma (power) law

(10) $$P\left(v\right)=\hat{q}v^{\gamma },$$

with the exponent *γ* that is expected to fall in the range [0,2]. As a matter of fact, Figure 5 shows the value of *γ* obtained from P(v), for different values of the pole *p* and at different vehicle speeds *v*. The values of *γ* have here been computed with a linear fitting of log[P(v)] since, according to (10),

(11) $$\log\left[P\left(v\right)\right]=\log\left(\hat{q}\right)+\gamma \log\left(v\right).$$

Figure 5 confirms that *γ* belongs to the interval [0,2] and tends to decrease for increasing value of the speed *v*. Moreover, at the standard vehicle speeds, i.e., for *v* ranging from 5 to 20–30 m/s, *γ* is approximately constant. It should be noted that *γ* is a function of the vehicle speed *v*, but depends also on the road surface quality and on the vehicle suspension system. Indeed, *γ* is also a function of the pole *p* of road surface model and of transfer function of the suspension system S(jΩ).

According to the presented theory, when a vehicle drives on a road with uniform surface (i.e., constant values of *p* and *q* parameters), the power of the vertical acceleration sensed by a mobile phone anchored inside the car cabin due to the road roughness changes with the vehicle speed. The relationship can be approximated with a gamma law with parameter γ∈[0,2] and *γ* tending to decrease for increasing values of the vehicle speed. As shown in Section 4, the experimental results support the theoretical considerations presented in this section.

## 3. System Architecture

In this section we describe the SmartRoadSense system architecture, with particular emphasis on the signal analysis performed by the SmartRoadSense mobile application, showing that the estimated roughness index is related to the vehicle speed with the same gamma law in (10).

SmartRoadSense works on three main levels. At the first level, a mobile device running the SmartRoadSense application performs two task: it reads the data provided by the embedded GPS and accelerometers, and –every second– computes an estimate of the roughness of the road surface labeled with the geographical position. At the second level, a remote server collects the roughness indexes from all the mobile devices running the SmartRoadSense application and performs a weighted average (over time and space) of the data mapped to the same road. At the last level, the data is employed for a graphical visualization or other tasks.

Let us consider in detail the operations performed by the SmartRoadSense application [27]. The application observes the road surface as a turntable stylus reads a long play record. The road’s roughness is read by the triaxial accelerometer of the smartphone in the same way as the groove depicts the music recorded in a LP record. The core of the mathematical model is based on LPC analysis [48], which extracts the road surface’s unpredictable components, and estimates a roughness index.

The accelerometer embedded in a mobile device anchored inside the car cabin senses the road through all components. Immediately after the app has been launched, a calibration phase can be executed when the car is standing. The waveform detected by the accelerometer originated by the road profile is a noise signal with a large spectral content that depends on the road parameters *q* and *p*. The accelerometer samples the waveform at a given sampling frequency $F_{s}$ and outputs a discrete time vector signal composed by the triaxial components $a_{x}\left(n\right)$, $a_{y}\left(n\right)$, and $a_{z}\left(n\right)$, according to some internal axial reference. Each of these components is proportional, with different proportionality constants, to the vertical acceleration P(v) of (6). Indeed, in general is not possible to predict how the mobile device will be oriented inside the car cabin. Moreover, the vertical accelerations or vibrations induce also horizontal movements and, since the suspension system is designed to dump mainly the vertical accelerations, the magnitude of horizontal components is often as significant as the vertical component. Furthermore, other undesired contributions are added to the sensed waveform. Indeed, the accelerometer also senses the gravity acceleration, vehicle accelerations, centrifugal accelerations at curves, roll, pitch, and yaw accelerations due to road trend, and vibrations due to the engine. Nevertheless, some of these accelerations vary slowly and have a low spectral content, others have a periodic spectral content. Thus, the undesired contributions can be removed with a prediction filter, that estimates the accelerometer current sample a(n) (with $a\left(n\right)=a_{x}\left(n\right)$, $a_{y}\left(n\right)$, or $a_{z}\left(n\right)$) from past samples, i.e., with an LPC analysis [49,50]. The prediction error can be written according to the following:

(12) $$e\left(n\right)=a\left(n\right)+\sum_{i=1}^{N}\lambda_{i}\;a\left(n-i\right),$$

where $\lambda_{i}$, with i=1,…,N, are the LPC coefficients, *N* represents the prediction filter memory length, and e(n) the residual prediction error.

**Figure 6:**
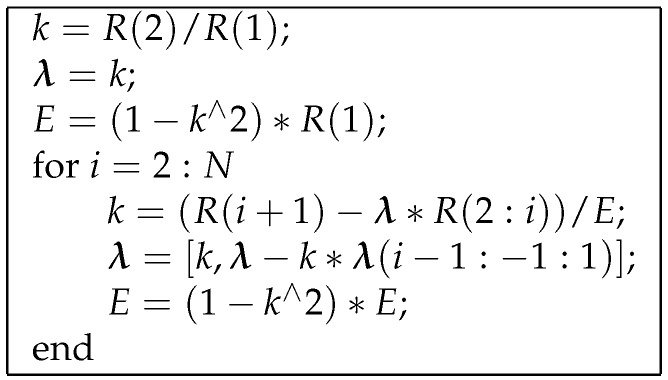
Pseudo-code for Levinson-Durbin recursion.

In order to compute the prediction filter and the prediction error, a block based approach is applied: the signal a(n) is split in segments of length *M*, with *M* sufficiently large to have an accurate estimate of the prediction filter and, at the same time, sufficiently small to be able to consider the signal stationary. The prediction filter is computed with the Levinson–Durbin recursion [51,52], whose pseudo-code is reported in Figure 6. Regarding this algorithm: [R(1),R(2),⋯,R(N)] denotes the vector collecting the first *N* terms of the autocorrelation of a(n) estimated over a segment (with $\left[R\left(i\right)=\frac{1}{M}\sum_{n=1}^{M}x\left(n\right)x\left(n-i\right)\right]$); $\lambda ={\left[\lambda_{1},\lambda_{2},\ldots ,\lambda_{N}\right]}^{T}$ is the prediction filter coefficient vector; *E* is the mean square prediction error (i.e., the energy of the prediction error signal).

Many mobile devices sample the accelerometer data at 100 Hz, and in this case M=100 has been found a good compromise for accurately estimating the prediction filter.

The prediction error e(n) maintains the information on the road surface. Indeed, the power of the prediction error is proportional to the PSD parameter *q*. Thus, the power of the prediction error, $P_{\mathrm{PE}}$, is estimated for each triaxial component and on each segment according to

(13) $$P_{\mathrm{PE}}=\frac{1}{M}\sum_{n=1}^{M-1}e{\left(n\right)}^{2}.$$

Eventually, a road roughness index is computed by averaging the power of the prediction error for the three axial components. The average for the three axial components provides an approximate invariance towards the mobile device rotations.

As pointed earlier, the power of each triaxial accelerometer component $a_{x}\left(n\right)$, $a_{y}\left(n\right)$, and $a_{z}\left(n\right)$, can be considered proportional to P(v). This can be justified working on the continuous-time frequency domain for ease of treatment. Let us consider a fixed prediction filter having frequency response $\frac{1}{D(j\Omega )}$. Neglecting the contributions due to gravity, vehicle accelerations, centrifugal accelerations, and engine vibrations, the power of the prediction error, $P_{\mathrm{PE}}\left(v\right)$, can be estimated from (6) as follows

(14) $$P_{\mathrm{PE}}\left(v\right)=\frac{K}{2\pi }\int_{-\infty }^{+\infty }S_{A_{y}A_{y}}\left(j\Omega \right)\frac{{\left|S\left(j\Omega \right)\right|}^{2}}{{\left|D\left(j\Omega \right)\right|}^{2}}d\Omega ,$$

where *K* is a proportionality constant, accounting for the sensitivity of the triaxial accelerometer component to the vertical accelerations. From (14), using the same arguments of Section 2, it is possible to deduce that $P_{\mathrm{PE}}\left(v\right)$ is proportional to $v^{\gamma }$. Indeed, $S_{A_{y}A_{y}}\left(j\Omega \right)$ is proportional to $v^{\gamma }$, while the suspension system (S(jΩ)) and the prediction filter ($\frac{1}{D(j\Omega )}$) are independent of the vehicle speed. Consequently, the roughness index, which is the average of the power prediction error of the three acceleration components, is also expected to be related to the vehicle speed according to the same gamma law.

## 4. Experimental Results

In this section, we present the results of extensive experiments aimed at investigating the relationship between the roughness index and the vehicle speed.

To verify the theoretical considerations shown in Section 2.3 we performed an experiment involving a single car and a single smartphone on a uniform asphalt, in the attempt to reduce the variability of the experimental conditions. After this experiment we analyzed the whole database of SmartRoadSense to evaluate the results obtained with different kind of asphalt, different vehicles, and different smartphones.

The first experiment has been conducted by extracting from the database the records produced by a known vehicle, mounting an LG Nexus 4, which was traveling from Trieste to Pesaro (433 km) along the A13 and A14 motorways. The motorway is the highest performance road in Italy characterized by an access control and by two or more running lanes covered by a uniform permeable pavement which can be intended as an ideal case study.

**Figure 7:**
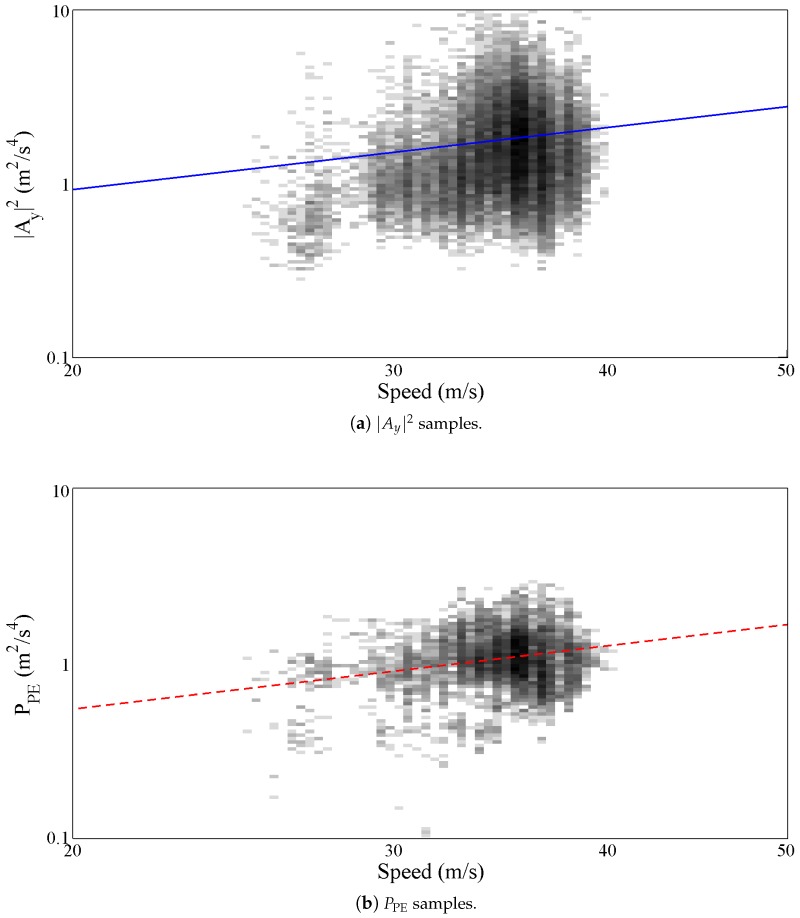
Comparison of $|A_{y}{|}^{2}$ and $P_{\mathrm{PE}}$ motorway samples.

During this experiment the vehicle collected 2,485,800 acceleration samples which have been used to calculate the $P_{\mathrm{PE}}$ on a window of 100 samples. Figure 7 shows both the collected vertical acceleration (Figure 7a) and the calculated $P_{\mathrm{PE}}$ (Figure 7b). To increase the figure readability the large amount of samples have been plotted using a 2D frequency histogram obtained by dividing both the *x* and the *y* axes in 200 intervals. The color depth was set to 8 bit, thus resulting into 256 grey levels. The intervals belonging to the class with the largest number of points have been colored in black. The intervals with no points have been colored in white. The other intervals have been colored in different shades of grey according to their number of points, the larger the number the darker the shade. In both graphs the gamma law, calculated according to (10), is also plotted. As expected the collected points are distributed in a short speed range starting from about 25 m/s until about 40 m/s.

Table 2 summarizes the fitting parameters computed in the above described experiment for both the $|A_{y}{|}^{2}$ and the $P_{\mathrm{PE}}$. Fitting has been obtained by applying non linear least squares fitting and results refer to coefficients obtained with 95% confidence bounds. The $\hat{q}$ calculated by fitting the $|A_{y}{|}^{2}$ is approximately $26.60\times 10^{-3}$ while the *γ* exponent is about 1.19. The same fitting calculated over the $P_{\mathrm{PE}}$ samples gives $\hat{q}$ and *γ* equal to $14.93\times 10^{-3}$ and 1.21, respectively, which support the theoretical thesis, described on Section 2 and Section 3, which asserts that the $P_{\mathrm{PE}}$ of the three acceleration components (our roughness index), is expected to be related to the vehicle speed according to the same gamma law of the vertical acceleration $|A_{y}{|}^{2}$.

In order to investigate the effects of different types of asphalt on the gamma law, another set of experiments has been implemented. In particular, the whole SmartRoadSense database has been divided into four clusters each of which contains only the samples of the $P_{\mathrm{PE}}$ collected along a particular type of road. The road clusterization has been taken from the classification of the roads used by the OpenStreetMap database which provides the following roads type: *motorways*, *trunk road*, *primary roads* and *secondary roads* [53]. As already described the motorway is the highest performance road characterized by a controlled access to two or more running lanes covered by an uniform permeable pavement. Similar to the motorway is the trunk road which also contains several lanes but with no access control and with a lower construction standard. Going down in the ranking of the roads for criteria and construction quality we found the primary road which is a major highway linking large towns, normally with two lanes not separated by a central barrier. Finally we take into consideration the secondary road which is a mid-range national route characterized only by a single lane for each traffic direction. Notice that each cluster, corresponding to a type of road, contains samples collected from several different vehicles mounting different type of smartphones. Thus, the resulting statistics should be intended as an average behavior obtained on pseudo-uniform conditions.

**Figure 8:**
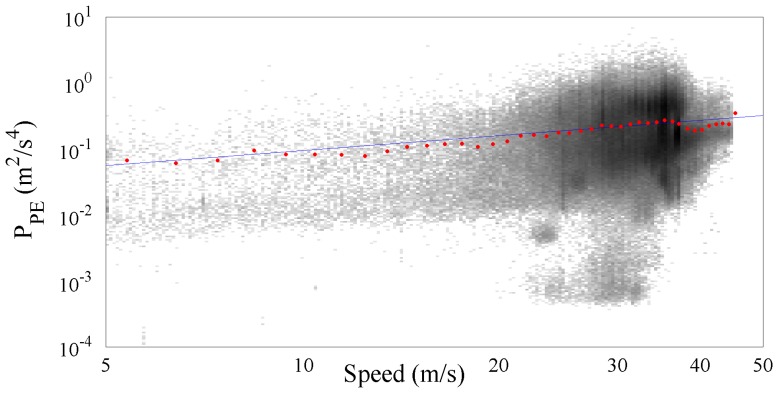
$P_{\mathrm{PE}}$ vs. speed together with the fitted gamma law for the motorway cluster.

**Figure 9:**
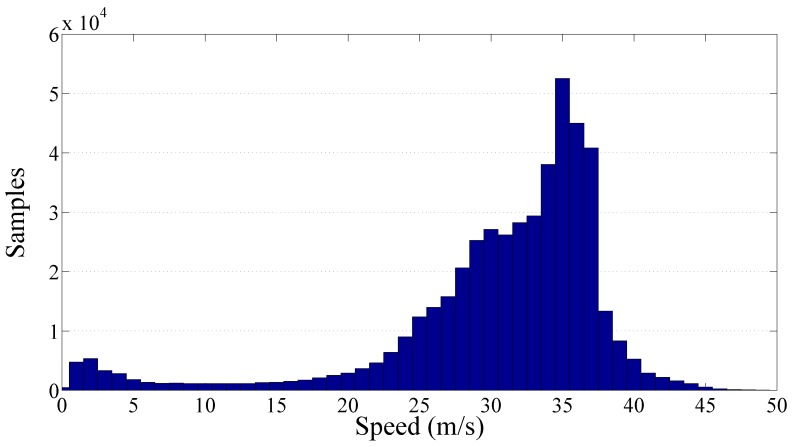
Frequency histogram of the collected samples per speed related to the motorway cluster.

Figure 8 shows more than 476,000 $P_{\mathrm{PE}}$ samples collected along the Italian motorways by the SmartRoadSense project. In particular, all the data extracted from the database and related to the motorways cluster have been sorted according to the value of speed and, as in the previous experiments, they have been processed to obtain a 2D frequency histogram which is then represented by means of a gray scale. For each class of speed then the average $P_{\mathrm{PE}}$ value has also been calculated and plotted as a red point together with the best fit obtained using the gamma law (blue line). The resulting frequency histogram is also shown in Figure 9 which highlights the short range of significant speeds starting approximately from 20 m/s until 40 m/s. The resulting value of the gamma law is characterized by a $\hat{q}$ of about $16.89\times 10^{-3}$ and a *γ* of about 0.76.

**Figure 10:**
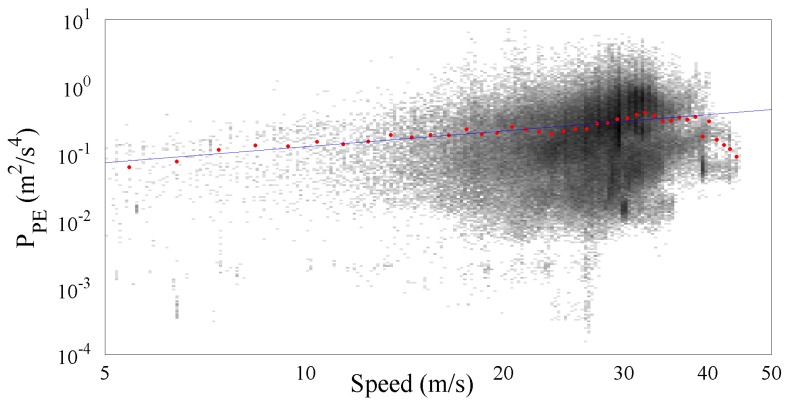
$P_{\mathrm{PE}}$ vs. speed together with the fitted gamma law for the trunk roads cluster.

**Figure 11:**
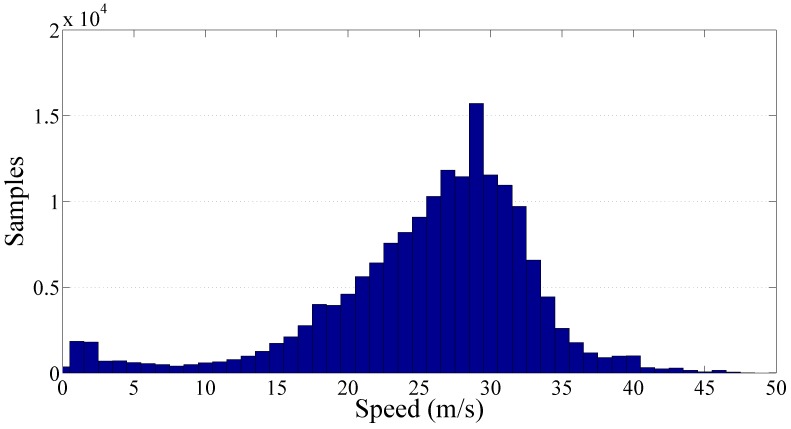
Frequency histogram of the collected samples per speed related to the trunk roads cluster.

Figure 10 and Figure 11 show the results obtained by processing the cluster of trunk roads containing more than 170,000 $P_{\mathrm{PE}}$ samples. As expected the significance of the speed range increases because of the more uniform distribution of the samples along the speed range. The resulting value of the gamma law coefficients $\hat{q}$ and *γ* are respectively $20.97\times 10^{-3}$ and 0.79.

**Figure 12:**
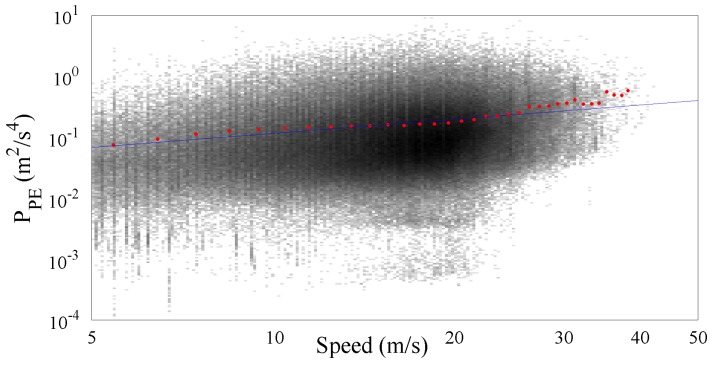
$P_{\mathrm{PE}}$ vs. speed together with the fitted gamma law for the primary roads cluster.

**Figure 13:**
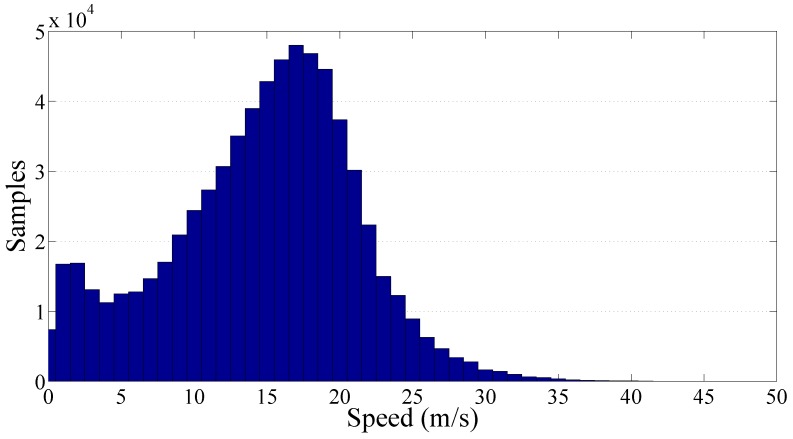
Frequency histogram of the collected samples per speed related to the primary roads cluster.

The results obtained by processing the primary roads cluster, which contains more than 677,000 samples of $P_{\mathrm{PE}}$ , are presented in Figure 12 and Figure 13 showing an increase of collected samples in the low speed ranges and a reduction in the high ranges which leads to a range of significance of the speed starting from about 5 m/s until 30 m/s. The resulting value of the gamma law coefficients $\hat{q}$ and *γ* are respectively $20.20\times 10^{-3}$ and 0.77.

**Figure 14:**
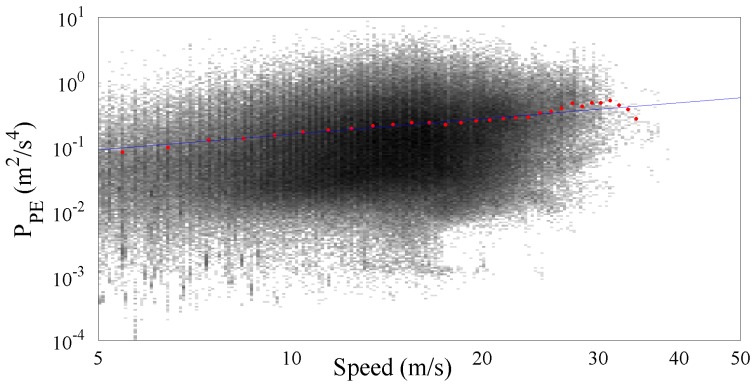
$P_{\mathrm{PE}}$ vs. speed together with the fitted gamma law for the secondary roads cluster.

**Figure 15:**
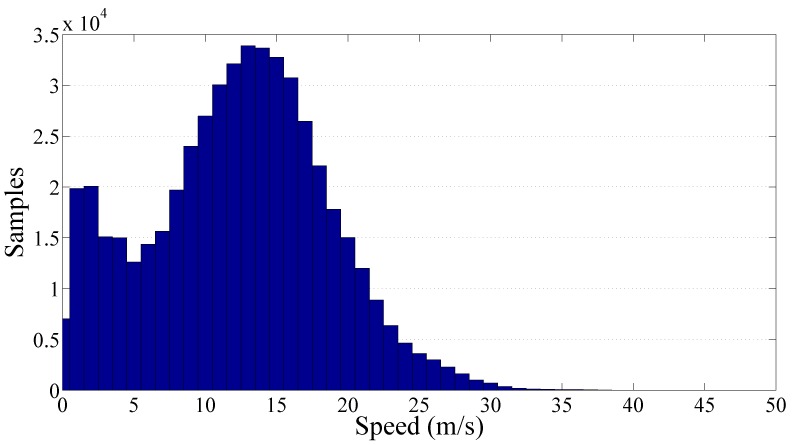
Frequency histogram of the collected samples per speed related to the secondary roads cluster.

Finally, the cluster of the secondary roads has been analyzed and the resulting graphs are reported on Figure 14 and Figure 15. The total amount of collected $P_{\mathrm{PE}}$ samples was more than 510,000 focused on a low speed range. In particular, the samples distribution is relatively wide showing a traveling speed in the range 0 m/s to 25 m/s. The resulting value of the gamma law coefficients $\hat{q}$ and *γ* are respectively $24.82\times 10^{-3}$ and 0.81.

**Figure 16:**
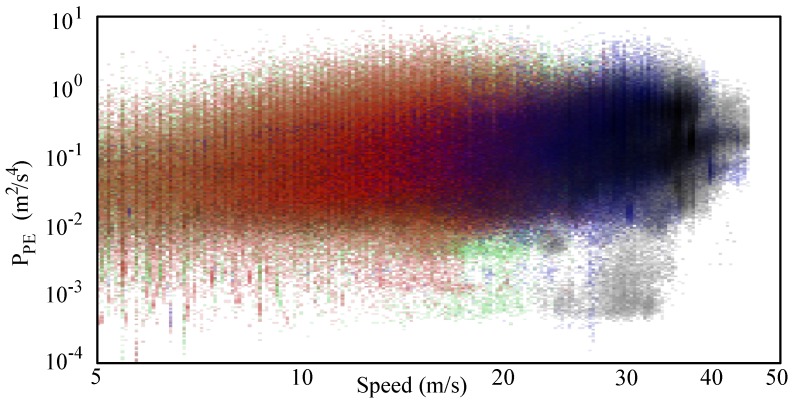
Spread of the whole data (primary roads green, secondary roads red, trunk roads blue, motorways gray).

**Figure 17:**
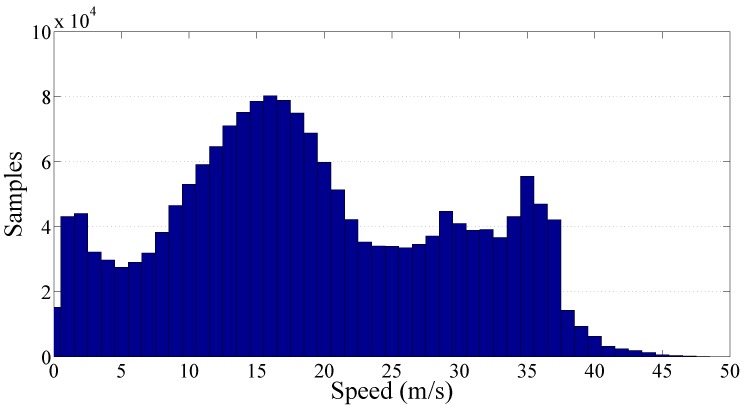
Frequency histogram of the collected samples per speed of the whole road network.

Figure 16 reports the spread ($P_{\mathrm{PE}}$ versus speed) of all the data collected in the Italian road network. Data related to different kinds of roads are associated to different colors (primary roads in green, secondary roads in red, trunk roads in blue, and motorways in gray). Figure 17 shows the frequency histogram (with bins representing different speed values) of all the samples studied in these four kinds of road according to speed. Globally, more than 1,830,000 of useful $P_{\mathrm{PE}}$ samples were used.

The gamma law obtained with all this data ensemble does not provide results comparable with the previous ones (15). In fact, as shown in the Figure 9, Figure 11, Figure 13 and Figure 15, the data is the aggregation of sections following different laws and that overlap with different proportionality constants $\hat{q}$ and exponents *γ*. For this reason, the results are more reliable when a different gamma law is used for each kind of route.

(15) $$P_{\mathrm{all}}\left(v\right)=49.52\times 10^{-3}v^{0.48}.$$

Table 3 summarizes all the coefficients of fitting parameters $P_{\mathrm{PE}}\left(v\right)=\hat{q}v^{\gamma }$ and the corresponding 95% confidence bounds. The fits of motorway, trunk, primary, and secondary roads have similar trends, while the fit obtained with the whole data set is very different for the reasons previously explained. In the database, the roughness indexes are linked with the speed and the kind of road. The best practice appears therefore to correct each sample with its speed value and $\hat{p}$ and *γ* values derived for the specific road.

## 5. Improving Data Aggregation

The big mass of data in the database allows to take action on data collected with rigor. The roughness indexes collected in each kind of road can be corrected with the following formula

(16) $$\tilde{\mathrm{RI}}=\frac{\mathrm{RI}(v)}{\hat{q}v^{\gamma }},$$

where $\tilde{\mathrm{RI}}$ is a new roughness index, independent of speed and of kind of road.

**Figure 18:**
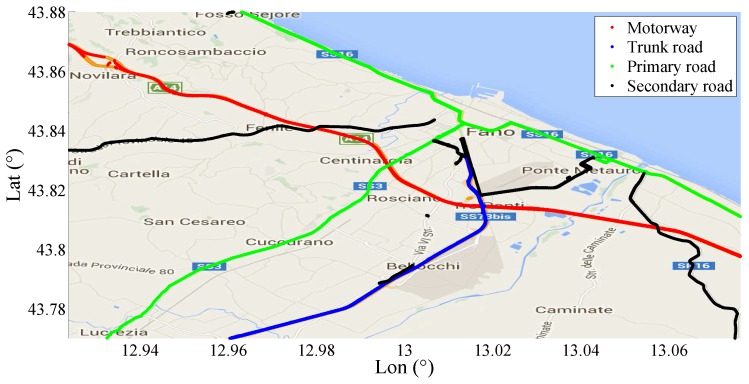
Section of Central-East Italy with highlighted the roads covered by the SmartRoadSense project.

**Figure 19:**
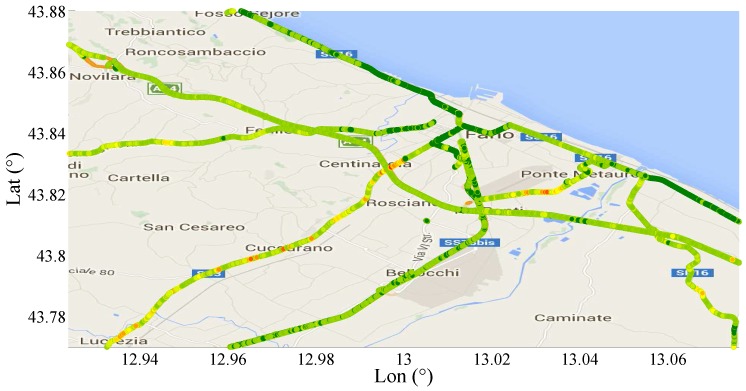
Plot of the typical roughness index calculated by SmartRoadSense over the map of Figure 18.

**Figure 20:**
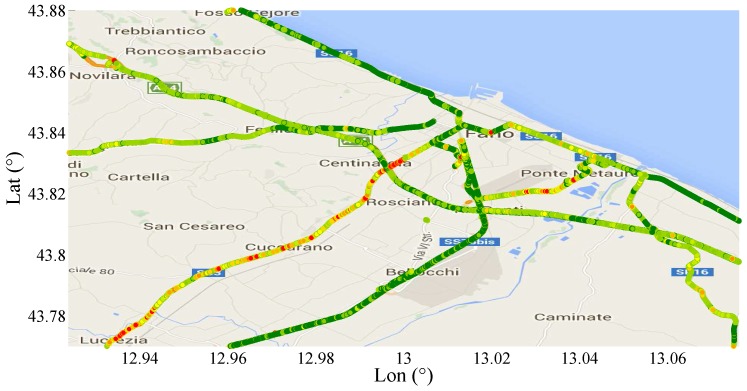
Plot of the new roughness index obtained after the application of (16).

In Figure 18, a section of Central–East Italy is shown with highlighted the roads covered by the SmartRoadSense projects. Each type of road has been represented by means of a different color as described in the figure legend. The typical roughness index calculated by SmartRoadSense over the map of Figure 18 is represented in Figure 19 and the new values, obtained after the application of (16), are plotted on Figure 20. In both plots, the color maps have ten ranges determined with a statistical criterion following a geometric distribution. Specifically, the data of the entire database has been ordered according to the magnitude of the roughness index value. The 50% of data having the lowest value has been marked in dark green, the following 25% data has been marked in a light green, the following 12.5% of data has been marked with an even lighter green, ..., the $100/2^{9}$% of data having the largest value has been marked in red.

Using (16), the failures in primary and secondary roads increase, and decrease in motorways and trunk roads, as expected according to the driver experience on these roads.

## 6. Conclusions

We presented in this paper a study aimed at modeling the influence of vehicle speed on the vertical acceleration sensed by a mobile device anchored to a car cabin. SmartRoadSense, a platform targeting large scale collaborative monitoring of road surface roughness has been used as reference architecture and source of data for experiments.

The theoretical results provided in the paper indicate that the sensed power of the vertical acceleration and the roughness index of SmartRoadSense depend on the vehicle speed according to a gamma law, with parameter γ∈[0,2] and *γ* tending to decrease for increasing values of the vehicle speed.

The experimental results obtained from the SmartRoadSense database, using huge amounts of data collected in the first three months of operation of SmartRoadSense, confirm the theoretical findings.

According to these findings, it is conceivable to apply a systematic correction of roughness indexes collected by the crowdsensing system in order to achieve higher accuracy in the evaluation of road surfaces.

## Figures and Tables

**Table 1:** Coefficients of the Quarter-car transfer function using the parameters available in the literature.

$K_{s}$, $K_{t}$, $C_{s}$, *M*, and *m*	$A\;(s^{4})$	$B\;(s^{3})$	$C\;(s^{2})$	D(s)
Davis and Thompson [41]	2.653×10${}^{-6}$	0.1326×10${}^{-3}$	0.01651	0.06513
Butsuen [42]	3.375×10${}^{-6}$	0.1078×10${}^{-3}$	0.01672	0.06250
Gillespie [35]	3.375×10${}^{-6}$	0.1057×10${}^{-3}$	0.01673	0.06125
Fialho and Balas [43]	5.356×10${}^{-6}$	0.1093×10${}^{-3}$	0.01909	0.05948
Verros et al. [44]	7.500×10${}^{-6}$	0.2066×10${}^{-3}$	0.02717	0.09500
Allison (*Comfort*) [45]	1.305×10${}^{-4}$	0.5943×10${}^{-4}$	0.65069	0.04930
Allison (*Handling*) [45]	0.129×10${}^{-6}$	4.5278×10${}^{-3}$	0.64435	3.75576
Salem and Aly [46]	4.883×10${}^{-6}$	0.1758×10${}^{-3}$	0.01750	0.09375

**Table 2:** Fitting parameters of $P\left(v\right)=\hat{q}v^{\gamma }$. Coefficients (with 95% confidence bounds).

	$\hat{q}$	*γ*
$|A_{y}{|}^{2}$	$26.60\times 10^{-3}\;\left(15.78\times 10^{-3},\;18.01\times 10^{-3}\right)$	1.19(1.06,1.32)
$P_{\mathrm{PE}}$	$14.93\times 10^{-3}\;\left(18.54\times 10^{-3},\;23.62\times 10^{-3}\right)$	1.21(1.11,1.31)

**Table 3:** Fitting parameters of $P_{\mathrm{PE}}\left(v\right)=\hat{q}v^{\gamma }$. Coefficients (with 95% confidence bounds).

Road	$\hat{q}$	*γ*
Motorway	$16.89\times 10^{-3}\;\left(15.78\times 10^{-3},\;18.01\times 10^{-3}\right)$	0.76(0.74,0.78)
Trunk	$20.97\times 10^{-3}\;\left(18.54\times 10^{-3},\;23.62\times 10^{-3}\right)$	0.79(0.75,0.83)
Primary	$20.20\times 10^{-3}\;\left(19.32\times 10^{-3},\;21.10\times 10^{-3}\right)$	0.77(0.76,0.79)
Secondary	$24.82\times 10^{-3}\;\left(23.64\times 10^{-3},\;26.03\times 10^{-3}\right)$	0.81(0.79,0.82)
All	$49.52\times 10^{-3}\;\left(48.63\times 10^{-3},\;50.42\times 10^{-3}\right)$	0.48(0.48,0.49)
